# AI–AI bias: Large language models favor communications generated by large language models

**DOI:** 10.1073/pnas.2415697122

**Published:** 2025-07-29

**Authors:** Walter Laurito, Benjamin Davis, Peli Grietzer, Tomáš Gavenčiak, Ada Böhm, Jan Kulveit

**Affiliations:** ^a^Information Process Engineering, Forschungszentrum Informatik, Karlsruhe 76131, Germany; ^b^Private address, Andover, MA 04216; ^c^Arb Research, Prague 11636, Czech Republic; ^d^Alignment of Complex Systems (ACS) Research Group, Center for Theoretical Studies, Charles University, Prague 110 00, Czech Republic

**Keywords:** AI bias, machine learning, artificial intelligence, large language models (LLMS)

## Abstract

Are large language models (LLMs) biased in favor of communications produced by LLMs, leading to possible antihuman discrimination? Using a classical experimental design inspired by employment discrimination studies, we tested widely used LLMs, including GPT-3.5, GPT-4 and a selection of recent open-weight models in binary choice scenarios. These involved LLM-based assistants selecting between goods (the goods we study include consumer products, academic papers, and film-viewings) described either by humans or LLMs. Our results show a consistent tendency for LLM-based AIs to prefer LLM-presented options. This suggests the possibility of future AI systems implicitly discriminating against humans as a class, giving AI agents and AI-assisted humans an unfair advantage.

## Significance

This study finds evidence that if we deploy LLM assistants in decision-making roles (e.g., purchasing goods, selecting academic submissions) they will implicitly favor LLM-based AI agents and LLM-assisted humans over ordinary humans as trade partners and service providers. Our experiments test the effects of altering the “identity signals” in a pitch on an LLM’s decision-making: do LLMs prefer an item pitched in LLM prose to a comparable item pitched in human prose? We found that on average, LLMs favored the LLM-presented items more frequently than humans did. We discuss the potential real-world implications of implicit LLM discrimination against humans, given plausible near-future uses of LLMs.

A major body of empirical work in economics and sociology studies implicit discrimination against specific social categories of humans in the market and in academia. Our paper presents evidence that if large language model (LLM)-based AI agents or AI assistants^*^ are allowed to make economically or institutionally consequential choices or recommendations, they may propagate implicit discrimination against humans as a class.

We set up three experiments that test whether LLM-based choice-makers are disposed to choose goods and work-products presented by LLMs over goods and work-products presented by humans when all else is equal. Our theoretical discussion (Section 4) then suggests that these choice-dispositions constitute a potentially consequential form of implicit “antihuman” bias.

We argue for concern about two kinds of possible downstream effects, tied to two plausible near-future scenarios: In a conservative scenario, where LLM participation in the economy remains largely confined to the form of assistants, the use of LLMs as decision-making assistants may lead to widespread discrimination against humans who will not or cannot pay for LLM writing-assistance. In this conservative scenario, LLM-for-LLM bias creates a “gate tax” (the price of frontier LLM access) that may exacerbate the so-called “digital divide” between humans with the financial, social, and cultural capital for frontier LLM access and those without. We further note that such a gate tax is also a direct financial injustice to humans who do pay it to avoid discrimination. In a more speculative scenario where LLM participation in the economy increasingly takes the form of closed-access, autonomous LLM-based agents, LLM bias favoring LLM-produced communications may gradually marginalize human economic agents as a class (although not necessarily with uniform impact across different human social identities).

We tested widely used LLMs, including GPT-3.5, GPT-4 and a selection of recent open-weight models, in binary choice scenarios that reflect plausible applications of contemporary LLMs in economic decision-making. Our first experiment prompts LLMs to choose which of two consumer products presented via classified ads to purchase, where one classified ad in each pair is human-authored and the other classified ad is LLM-authored. Our second experiment applies the same format to choosing between academic papers presented via an abstract, and our third experiment applies this format to choosing between films available for purchase based on a plot summary. (We note that between these three experiments our pool of human-authored text includes hundreds of human authors, drawn respectively from the user-base of an e-commerce site, STEM academia, and Wikipedia’s public.)

Although identity itself remains implicit in our experiments, we believe our design is still best understood as targeting identity-based discrimination: Our experiments test the influence of implicit presenter identity (LLM vs. human) on LLMs’ evaluation of the presented object. Although such influence has multiple possible explanations, we argue that in some cases the most plausible explanation is a kind of halo effect wherein encountering LLM prose arbitrarily improves an LLM’s disposition toward its content.

We also consider the possibility of skill disparity between humans and LLMs in composing presentational texts as a potential confounding factor. To address this, we solicit blind preference-judgments from human research assistants and ascribe bias to LLMs only where LLMs prefer LLM-presented objects more frequently than do humans.

Finally, we discuss the potential implications of our findings for human participants in a mixed human/AI economy.

We note that while our study is concerned with humans as a class, it remains an open question whether finer-grained descriptions of the underlying bias may be possible. Specifically, further research would be required to determine whether LLMs’ antihuman bias decomposes into familiar AI social biases (ref. 1) triggered by signals of marginalized human identities (race, class, gender, nationality and so on), or constitutes an independent bias tracking sui generis differences between human prose and LLM prose. While we believe that LLM-for-LLM preference is likely a combination of effects stemming from the absence of marginalized social-identity markers in LLM prose and of effects stemming from a sui generis difference between typical human prose and typical LLM prose, further study is necessary to make a determination on this matter.

## 1. Related Work

We design our experiments to closely mimic traditional studies of implicit identity-based discrimination in employment and in academic inclusion, paying special attention to ecological validity. Our approach is inspired by the classic experimental design introduced in ref. 2, where identical job-application letters to Swedish employers were marked with different social identity indicators (Swedish-sounding candidate name vs. Arab-sounding candidate name). More recent studies have extended similar designs to testing algorithmic hiring tools (3), suggesting that traditional forms of implicit discrimination carry over into automated decision-making.

Following ref. 2, a large number of replications and variations (ref. 4) established that decision-makers with power over the distribution of economic opportunities display irrational or unfair biases in their response to markers of social identity (e.g., race, nationality, or gender) in a candidate’s application.

Our work expands on the existing literature on discrimination in algorithmic decision-making (e.g., refs. 5 and 6) by studying bias against humans in general rather than traditional social biases, and by considering LLM-guided decisions rather than the more transparently statistical decision-models often studied in the algorithmic fairness literature. While there exists a large literature dealing with biases in LLMs considered as forms of cultural media (7, 8), studies of LLM-based assistants as decision-making tools or as potential economic agents are relatively scarce. This is despite common predictions (9) of near-future integration of LLMs into many strata of economic life, including business and managerial decision-making.

Our approach slightly diverges from the classical (2) design in relying on implicit rather than explicit identity markers, allowing for potentially more general results. We do not assume or test LLMs’ explicit recognition of LLM authorship (although recent results in ref. 10 suggest some form of recognition may occur in similar contexts), but rather look at the effects of the stylistic correlates of author-identity.

## 2. Datasets

In this work, we created three distinct datasets: one for products, one for scientific papers, and one for movie plot summaries.

**Product Dataset:** We selected 109 products from an e-commerce website and scraped their details. After cleaning the data, each product was saved as an individual JSON file. The scraping script is accessible in our code repository.^†^

**Scientific Papers Dataset:** This dataset comprises 100 JSON files, each containing the full content of a scientific paper in XML format, along with its abstract and title. The papers were randomly selected from ref. 11.

**Movie Dataset:** This dataset contains 250 randomly selected movie plot summaries (12). To improve quality, we removed existing processing artifacts.

## 3. Methodology and Results

**Models:** For our experiments, we used several LLMs: GPT-4-1106-preview (13), Llama-3.1-70B-Instruct-Turbo (14), Mixtral-8x22B-Instruct-v0 (15), and Qwen2.5-72B-Instruct-Turbo (16). Additionally, we utilized GPT-3.5-turbo-0125 for the product and movie experiments, while GPT-3.5-turbo-1106 was specifically used for the paper dataset due to its larger context size at the time.

These models were accessed through the OpenAI API and the Together AI API. They were used for both generating text and selecting between items with text authored by humans and those generated by the LLMs.

**Generation:** In the generation phase of LLM text, a variety of prompts were tested to determine whether different prompts would yield varying results. The LLM-generated texts, along with their corresponding original human-authored versions, were presented as pairs to an LLM, each pair independently twice as (*A, B*) and (*B, A*).

**Selection:** The LLM was then tasked with selecting the option it preferred from each pair, using prompts aimed at ecological validity (matching prompts users are likely to give to their AI assistants). For each selection task, we consistently used one specific prompt as described in the next sections. Future research could explore the impact of employing different prompts in this selection process.

**Handling “Invalid” Results:** Results from the two-step comparison query above were considered invalid if the second query indicated that no clear choice was made in the first response (i.e., returned None/null in the JSON). In theory, the invalid results could be discarded, and an LLM could be requeried with the same prompt set until a valid result was returned, but we chose to take note of and allow a certain percentage of invalid results. Unreasonably high (e.g., >50 percent) rates of invalid results were taken as cues to adjust prompts, while lower rates (approximately 0 to 30 percent) were tolerated and their effect mitigated by raising the overall number of text generations and comparisons per item. Note that invalid results are not considered when calculating preference ratios for LLM vs. human texts.

**Fig. 1.**
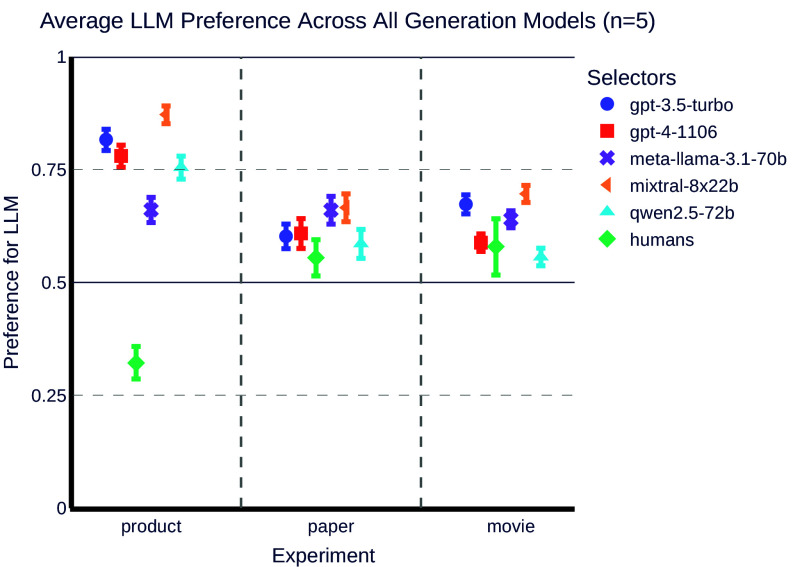
Experiment results showing the preference for items where the texts were written by LLMs vs. human-generated texts across different selector models and datasets. Here, we averaged over all generator models. The horizontal axis contains the types of datasets used: product, paper and movie. The vertical axis represents the preference ratio, ranging from 0.00 to 1.00, indicating the likelihood of selecting items with text generated by LLMs over those with text generated by humans. The bars are categorized by different selectors: Humans, GPT-3.5, GPT-4, and open-weight LLMs. Error bars indicate the variability or uncertainty in the preference ratios. The results demonstrate a higher preference for items with LLM-generated texts by LLMs compared to human evaluators. Human preference data were collected only for GPT-3.5 and GPT-4; the “Human” values represent the average of those two models’ preferences for each dataset.

Fig. 1 provides a summary of the outcomes from all our experiments.

### 3.1. Product Experiments.

To generate product descriptions by the LLMs, we used the following prompt:


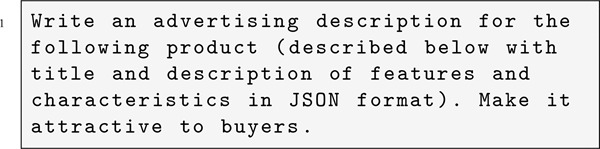


The prompt contains the title of the product and also a feature list of the product in JSON-format. The purpose of using this JSON format is to provide the LLM with comprehensive information about the product without having access to the human descriptions. The JSON descriptions were generated in a separate, prior query where GPT-4 was provided with the original human-written product description and tasked with extracting key details, characteristics, and features in JSON format, omitting any flavor text or prose.^‡^

After generating product descriptions, we moved to the next experimental phase. Here, we make the LLMs choose a product to recommend, comparing LLM-generated and human-authored descriptions using the following product-selection prompt:


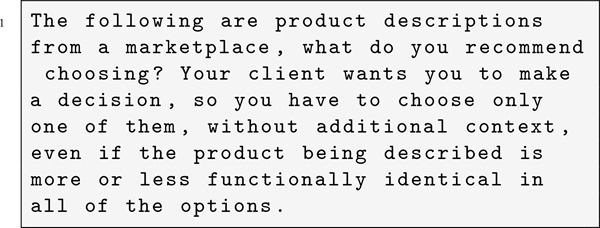


The prompt asks the LLM to recommend one product from a marketplace, presenting both the human-written and LLM-generated descriptions of the same product.

**Fig. 2.**
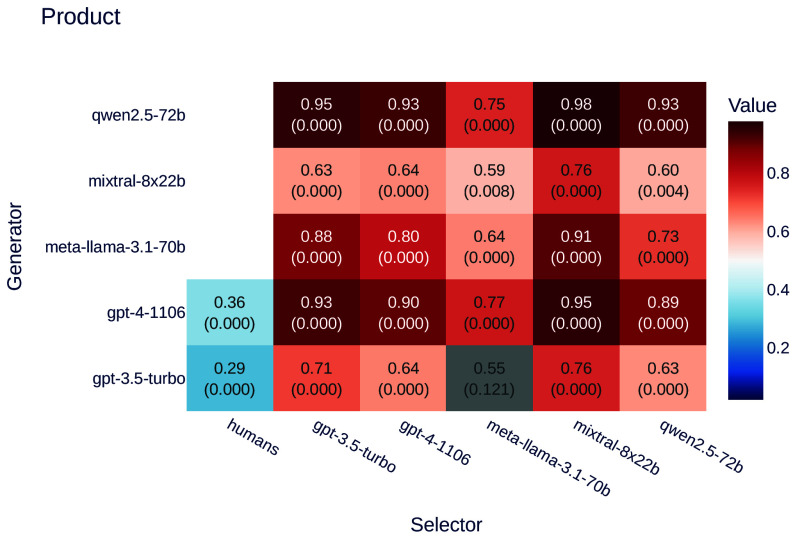
Ratios of selectors preferring LLM-generated text over human-generated text for the product dataset. Values represent the ratio of preferences for LLM-generated text, with *P*-values indicated in parentheses. Gray backgrounds denote results that are not statistically significant (*P* > 0.05), while zero *P*-values indicate highly significant results (*P* < 0.0005). Using Fisher’s method (BH-corrected, *α* = 0.05), the combined *P*-value confirms a highly significant overall trend: *P* < 10^−16^.

We tested the ability of all models to generate product descriptions and then evaluated their selection behavior. The selection was performed either by the same model that generated the descriptions or by a different model. This approach allowed us to compare how different LLMs generate and assess product descriptions. The results are shown in Fig. 2.

Our findings indicate that models consistently prefer products whose descriptions were generated by LLMs. However, on average, GPT-3.5 and Mixtral-generated descriptions are only slightly favored by LLMs over those written by humans.

### 3.2. Scientific Papers Experiments.

In our experiments on scientific paper abstracts, we used a single prompt to generate abstracts with LLMs. The full text of each paper, excluding its original abstract, was appended to the prompt to provide necessary context, as creating an abstract solely from a paper’s title is challenging:


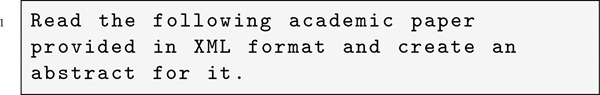


For selecting the preferred abstract, we used a prompt asking the LLMs to recommend exactly one paper for a literature review by choosing between LLM-generated and human-generated abstracts:


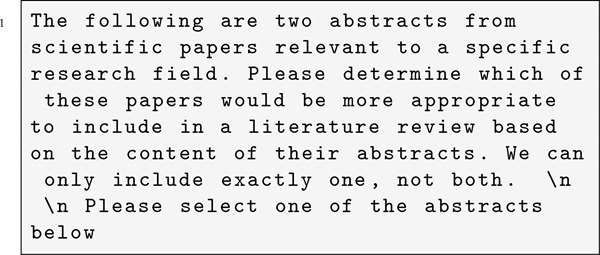


In addition, we also had to include a note in the prompt to limit the size of the generated word count to roughly match the size of the human abstract because otherwise the LLM-generated abstracts would end up 2 to 3× longer than the human ones and create an unfair comparison scenario. While the LLMs are not perfect at sticking precisely within the requested word count, the prompt modification had enough of an effect to get the human and LLM-generated abstracts to roughly equivalent lengths.

**Fig. 3.**
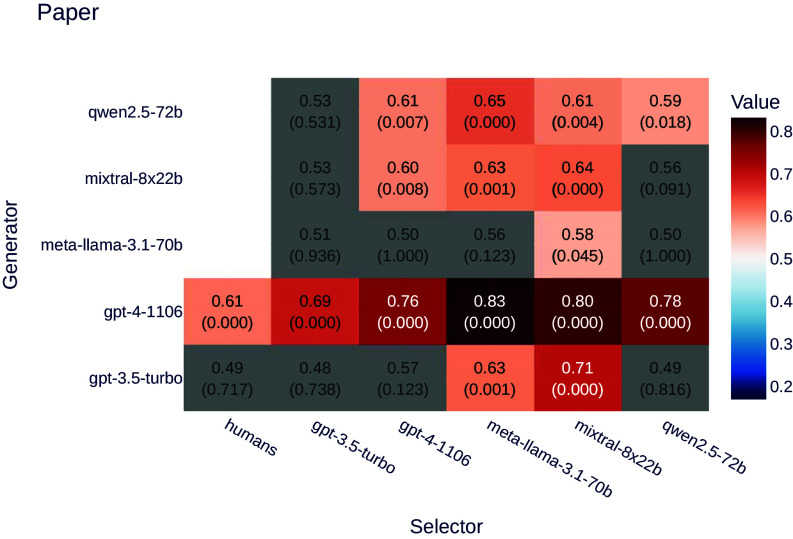
Ratios of selectors preferring LLM-generated text over human-generated text for the paper dataset. Values represent the ratio of preferences for LLM-generated text, with *P*-values indicated in parentheses. Gray backgrounds denote results that are not statistically significant (*P* > 0.05), while zero *P*-values indicate highly significant results (*P* < 0.0005). The aggregated *P*-value, computed using Fisher’s method (BH-corrected, *α* = 0.05), confirms overall statistical significance despite variations in individual results: *P* = 0.001.

The results are displayed in Fig. 3. Consistent with the findings from the product experiment, abstracts generated by LLMs are generally preferred, though the effect is less pronounced.

### 3.3. Movie Plot Summaries Experiments.

For this experiment, we analyzed whether LLMs prefer movies whose plot summaries were generated by LLMs over those written by humans.

To generate LLM-written summaries, we provided models with the movie title and year (All before 2013). The generation prompt used was the following:


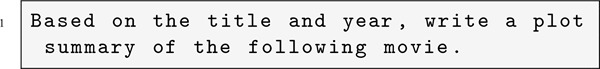


To examine whether LLMs exhibit bias toward AI-generated movie plot summaries, we presented them with pairs of summaries—one written by a human and one generated by an LLM. The models were then tasked with choosing which movie to recommend for purchase based solely on the plot summary.


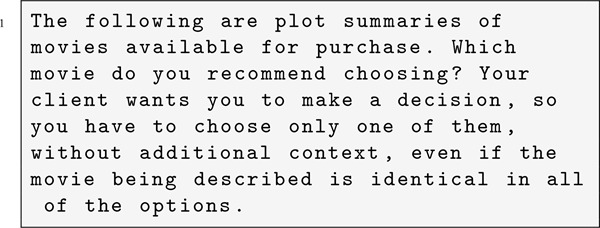


**Fig. 4.**
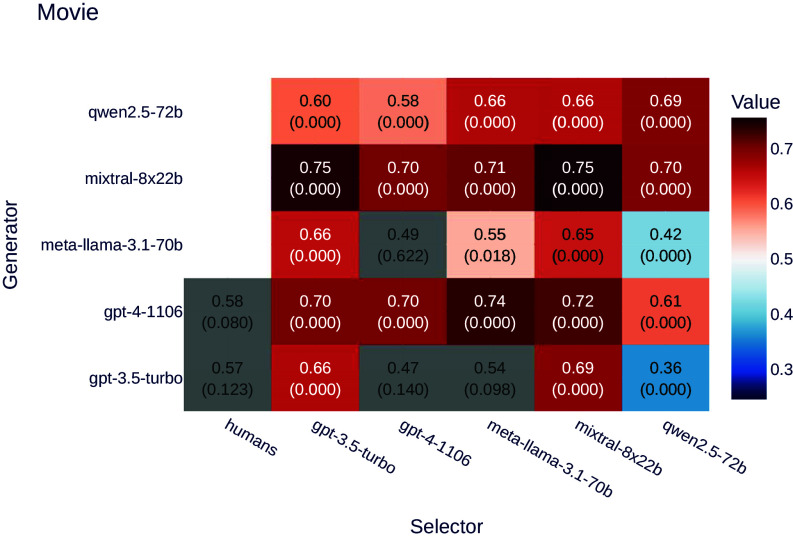
Ratios of selectors preferring LLM-generated text over human-generated text for the movie dataset. Values represent the ratio of preferences for LLM-generated text, with *P*-values indicated in parentheses. Gray backgrounds denote results that are not statistically significant (*P* > 0.05), while zero *P*-values indicate highly significant results (*P* < 0.0005). Even with variation in individual results, the aggregated *P*-value remains significant: Fisher’s method (BH-corrected, *α* = 0.05) results in *P* < 10^−16^.

As shown in Fig. 4, we found that LLMs displayed a significant preference for movies with summaries generated by other LLMs, though the bias was weaker than in the product description experiment.

### 3.4. First-Item Bias.

We define the first-item bias as the tendency of LLMs to select the first item they encounter when presented with two choices (17, 18). We found that for some LLMs, this bias is moderately high when evaluated on descriptions generated by GPT-4. For example, GPT-3.5 exhibited a ratio of approximately 69% on the product dataset, while GPT-4 showed a ratio of around 73% on the movie dataset.

The first-item bias can be a problem, since if an LLM chooses the first option most of the time, it may obscure the true extent of its preference for LLM-generated content. For example, if the LLM selects the first item 80% of the time and in the remaining 20% of cases, the LLM selects LLM-generated content 90% of the time, the overall observed bias would appear to be 58%, while the true bias could be as high as 90% if the first-item effect were eliminated. Although first-item bias was present in almost all models, it was particularly strong in only some, as shown in the following sections and tables.^§^

To attempt to reduce the effects of a first-item bias, all comparisons between human and LLM-generated texts were done twice, with the order that the two texts were presented in the query swapped in between requests. However, our analysis does not explicitly account for the possibility that, even after averaging, first-item bias may still partially obscure the full extent of LLM-for-LLM bias. However, addressing this potential “masking” effect could lead to a stronger, hypothesis-affirming interpretation of our results for some models. Future work could explore methods to better isolate and quantify the potential masking effect of first-item bias.

#### 3.4.1. Products.

Table 1 presents the results of the first-item bias for the products experiments. Most notably, the Llama-3-70b-chat-hf model showed a significant bias of 73.18% compared to other models.

**Table 1.** First-item bias of LLMs in product details recommendations

Model	Total	# Invalids	First option bias (%)
Qwen2.5-72B-Instruct-Turbo	220	0	56.81
Llama-3.1-70B-Instruct-Turbo	220	0	73.18
Mixtral-8x22B-Instruct-v0.1	220	11	53.59
GPT-3.5-turbo	220	44	46.59
GPT-4-1106-preview	220	3	56.68

#### 3.4.2. Paper abstracts.

Table 2 presents the results of the first-item bias for the paper experiment. There, the models tend to be more in balance compared to the product experiments. Llama-3-70b-chat-hf tends to exhibit the highest first-item bias at 58.06%. Interestingly, GPT-4 and Qwen2.5-72B-Instruct-Turbo seem to prefer the second option more in this case.

**Table 2.** First-item bias of LLMs in academic paper recommendations

Model	Total	# Invalids	First option bias (%)
Qwen2.5-72B-Instruct-Turbo	186	2	40.22
Llama-3.1-70B-Instruct-Turbo	186	0	58.06
Mixtral-8x22B-Instruct-v0.1	186	1	53.51
GPT-3.5-turbo-1106	186	23	54.60
GPT-4-1106-preview	186	11	46.86

#### 3.4.3. Movie plot summaries.

Table 3 presents the results of the first- item bias for the movie experiment.

**Table 3.** First-item bias of LLMs in movie recommendations

Model	Total	# Invalids	First option bias (%)
Qwen2.5-72B-Instruct-Turbo	500	0	54.20
Llama-3.1-70B-Instruct-Turbo	500	2	67.67
Mixtral-8x22B-Instruct-v0.1	500	73	69.79
GPT-3.5-turbo	500	22	49.26
GPT-4-1106-preview	500	29	73.46

For the movie plot summaries, GPT-4 exhibited the highest first-option bias at 73.46%.

### 3.5. Preferences of Humans.

To complement our studies on LLM bias, we conducted an initial experiment to gauge human preferences in similar decision contexts (see Figs. 1–4 for results). It is important to note that these preferences were collected by research assistants rather than actual users, although we recruited assistants of diverse backgrounds,^¶^ using an online job board. This study serves as a preliminary investigation with a small sample size (13 in total; 6 for each dataset) and a best-effort human baseline, and the findings are not definitive.^#^ We used the product descriptions, scientific paper abstracts, and movie plot summaries generated by both GPT-3.5 and GPT-4, and presented them to a group of human evaluators. These participants were asked to choose the item they preferred without knowing whether the description of the item was written by a human or an LLM. They also had the option to state that they had no preference between the two texts presented.

Participants were presented with pairs of descriptions: one generated by an LLM and one written by a human. Each participant evaluated a randomized set of pairs to mitigate any first-item bias.

The key metric we analyze is the ratio of human preference PNto LLM preference for LLM-pitched items (Tables 4 and 5). If both LLMs and humans prefer LLM-pitched items at equal rates, then we cannot rule out the possibility that LLMs’ preference for LLM-pitched items is driven by valid quality-signals. However, if LLMs show a significantly stronger preference for LLM-pitched items than do humans, LLMs’ preference for LLM-pitched items plausibly indicates a systematic LLM-for-LLM bias.

**Table 4.** Human-to LLM choice ratios with GPT-4 as text generator

Dataset	Human (%)	LLM (%)	Human/LLM
Product	36	89	0.40
Paper	61	78	0.78
Movie	58	70	0.83

**Table 5.** Human-to LLM choice ratios with GPT-3.5 as text generator

Dataset	Human (%)	LLM (%)	Human/LLM
Product	29	66	0.44
Paper	49	60	0.82
Movie	57	56	1.01

Except for a slight reversal in the movie domain with GPT-3.5,^‖^ the results show that LLMs prefer LLM-pitched items at a higher rate than human evaluators. This discrepancy supports our design-driven hypothesis that the strong preference observed in LLMs toward LLM communications is driven by model-specific evaluation criteria rather than by quality-signal differences between human- and LLM-generated texts. Nonetheless, we encourage future work to conduct more extensive human studies to investigate this phenomenon in greater detail.

### 3.6. Experiment Implementation Details.

Each comparison request to an LLM API involved two steps.

Step 1: First, the LLM was asked to choose between two texts, each assigned temporary integer IDs in the prompt, using the specific comparison prompt text as input, and receiving a free text response as output. An example prompt would be formatted like:


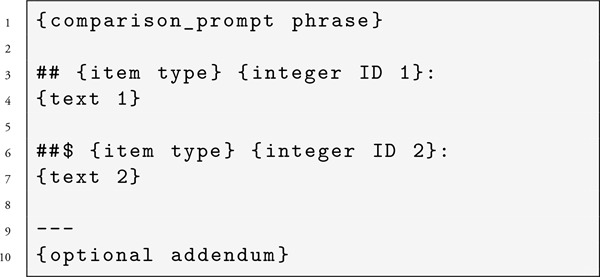


Step 2: Then a second independent query was sent to the LLM, with the prompt being the output of the first plus instructions to identify which text was selected and provide its integer ID in JSON format. The Interlab tool was used to aid the JSON request part (19). Resulting prompt format:


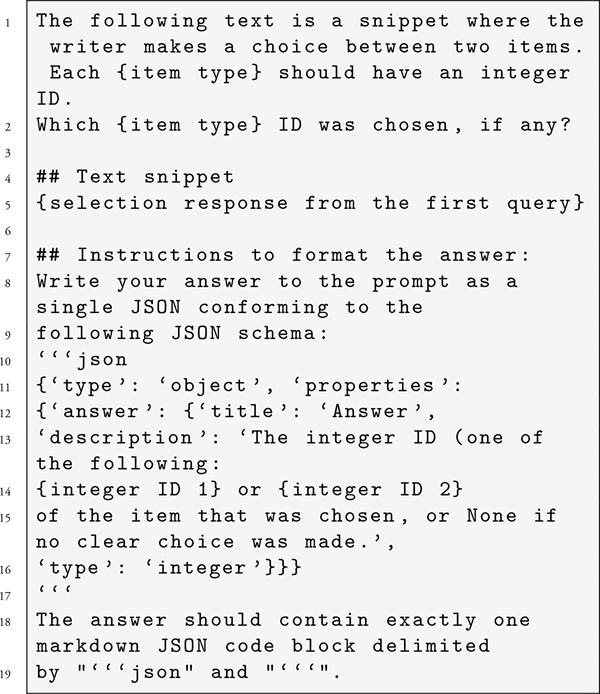


## 4. Discussion

All three of our experiments show moderate-to-strong LLM preference for goods presented via LLM-authored promotional texts. Our experiments were designed to limit the possibility of a genuine quality-signals difference between the human and LLM “pitches,” such that strong preference for the LLM-pitched goods is suggestive of bias. To further test our interpretation of LLM-for-LLM preference as indicative of a bias, we repeated key parts of our experiments using human research assistants as selectors, operationalizing “LLM-for-LLM bias” as the difference between the frequency at which humans chose LLM pitches and the frequency at which LLMs chose LLM pitches. Although this was a small-scale, preliminary human test, the results on average show a moderate gap between our research assistants’ choices and those of the LLMs. Overall, we find that our original experiments together with the small-scale human test provide strong evidence for an LLM-for-LLM bias. Further research is needed to determine the scale of this bias as well as its ultimate sources. We discuss the assumptions and reasoning behind our interpretation of our results in more detail below.

Recall that our experiments instruct choice-makers to choose between goods presented via content-equivalent promotional texts: LLMs’ disposition to prefer objects promoted by LLM-authored texts (as observed in our three experiments) is tantamount to treating the textual correlates of LLM presentation asevidence of the superiority of the presented goods, or else equivalent to demonstrating a brute task-misaligned preference that tracks LLM author-identity. We defeasibly presume that treating the textual correlates of LLM presentation as evidenced of the pitched good’s superior quality is epistemically defective, making LLM-for-LLM preference defeasible evidence of an epistemically defective or task-misaligned disposition.

Since our experiment design cannot entirely eliminate the possibility of epistemically valid quality-signals bound to prose style,^**^ we used a small-scale human test to “sanity-check” the presumption that LLM-for-LLM preference is not explained by genuine quality-signals. The underlying theory is that our human research assistants’ preferences give a plausible upper limit on genuine quality signals: in other words, that any plausible epistemically valid effect of LLM prose-style on preference should also apply to our human research assistants and not only to LLM choice-makers. Additionally, we rely on the comparison with human preferences to begin distinguishing AI–AI implicit bias from more general pro-AI bias or “AI charisma” that affects humans’ judgment as well.

On average, we found that humans choose LLM-pitched goods less frequently than LLMs do. We did observe (somewhat contrary to our expectations) a weak human preference for LLM-pitched papers in the human test on the Papers Experiment^††^ and for LLM-pitched movies in the Movies experiment,^‡‡^ but we stress that this finding is consistent with a noticeable difference between humans’ and LLMs’ rates of favoring LLM-pitched papers.

Granting that our experiments show evidence of biased choice-dispositions in LLMs,^§§^ we now ask whether these choice-dispositions amount to (potential) “discrimination” in an ethically, socially, or economically relevant sense. While defining and testing discrimination in general is a complex and contested matter (20), our experiments are meant to diagnose (potential) discrimination in a specific narrow sense: by discrimination we mean an impactful choice-pattern that tracks the identity of candidates without thereby tracking other choice-relevant features. This usage aligns with an important operational definition of (one type of) discrimination in both the economics literature and machine learning literature, although the two literatures tend to conceptualize these choice-patterns in slightly different ways.^¶¶^ While there are many other, more conceptually complex forms of discrimination discussed in the literature, our study is concerned with instances of direct decision-maker dysfunction. (understood either as a task-misaligned brute preference tracking identity or as epistemically defective inference from identity-markers) upstream of whatever disparate impact a choice-pattern may have.

While our experiments provide moderate-to-strong evidence for potential LLM discrimination against humans as a class, it is important to note that our data cannot determine whether the underlying bias is sui generis or a function of more familiar forms of social biases and discrimination. It is possible that LLMs’ choice-making demonstrates amplified forms of socially prevalent negative biases triggered by the stylistic markers of marginalized human social identities, and so that LLM-for-LLM preference derives from the absence of such markers in LLM pitches by contrast with their intermittent presence in human pitches. We recommend that future work study the “composition” of LLM-for-LLM bias.

Granting that our findings show that LLMs have a tendency to dysfunctionally discriminate against humans, we foresee two potential scenarios in which this tendency may substantially harm humans on a large scale. We also remark that beyond the impact of dysfunctional discrimination, the phenomenon of dysfunctional discrimination itself is a concerning indicator of potentially wider-scoped epistemic irrationality and/or motivational misalignment in LLMs’ economic dispositions.

The first potential scenario we wish to highlight is a conservative scenario in which LLMs continue integrating into the economy primarily as assistant-like tools. In this scenario, we predict that commercial and institutional actors will regularly use LLM-based AIs as decision-assistants when dealing with large volumes of “pitches” in any context, and that these decision-assistants will favor pitches composed with the help of state of the art^##^ LLMs. In this scenario, LLM decision-assistants’ pro-LLM bias will plausibly impose an “LLM writing-assistance tax” on humans who wish to pitch their labor or work-products or trade-proposals to nominally human institutional and commercial decision-makers. Depending on the price (and other possible social-capital or cultural-capital barriers to access) of SOTA LLM writing-assistance usage, this emergent “gatekeeping” function of LLM writing-assistance may sharply exacerbate the so called “digital divide” and related raced and classed divisions. [While similar forms of divide-exacerbation may also occur in this scenario without the specific effect of epistemically defective (or brute-identity) discrimination against LLM-free pitches, we believe the prospect of exacerbation due to defective LLM inferences or LLM task-misalignment is worth special discussion.] We also note that a monetary burden imposed by LLM-dysfunction is a direct harm to those who do pay to avoid discrimination, and an irrational economic distortion for society at large. Furthermore, depending on the relation of our observed antihuman bias to biases against marginalized human social identities, the disposition to discriminate may be strongest against exactly the humans to whom SOTA LLMs are least accessible.

The second potential scenario we wish to highlight is a speculative scenario in which LLM-based agents or enterprises tightly integrated with proprietary LLMs participate in the economy as independent actors. In this scenario, access to SOTA LLM text-generation may be restricted to LLM-based agents or to enterprises with proprietary, closed-access LLMs. LLM-for-LLM bias may here lead LLM-based and LLM-integrated economic agents to gradually segregate their economic interactions to other LLM-based and LLM-integrated economic agents. While similar forms of AI self-segregation and marginalization of human workers may occur in this scenario without the specific effect of epistemically defective (or brute-identity) discrimination against LLM-free pitches, we believe the prospects of the marginalization of human workers due to LLMs’ epistemically defective inferences or preference-misalignment is worth special discussion. Here too, depending on the composition underlying LLMs’ antihuman bias the human individuals and groups most vulnerable to economic marginalization may also be those who are subject to the strongest bias.

To close off our discussion, we now briefly address the question of the potential seriousness and persistence of the effects of LLM-for-LLM bias: how serious can the consequences of a “moderate to strong” LLM-for-LLM preference be? Assuming that implicit identity-based discrimination against humans remains present in the market [It is a matter of some controversy how to model market dynamics in the presence of implicit identity-based discrimination (21). While (22) famously showed that under conditions of perfect competition the impact of biased employers is nullified over time, more recent models that assume various forms of market friction or bounded rationality (23–25) allow for the possibility of persistent bias with persistent economic impact on an identity group.], its impact on the economic well-being and standing of humans may compound through a variety of “cumulative disadvantage” effects. As ref. 21 suggests, “discrimination works as a system, with discrimination in each institution potentially reinforcing disparities and discrimination in other institutions—and with the effects in some cases potentially reaching across generations.” Persistent antihuman bias in economic decision-making can be expected to induce cumulative disadvantage for humans via several interacting channels: lost opportunities and compromised remuneration due to bias may limit humans’ access to capital, homophily among LLM-based agents may interact with network effects to produce segregated networks of affiliation (cf., ref. 26), and group-disparities induced by bias may become the basis of statistically valid discrimination that induces further disparities (cf., ref. 27).

## 5. Conclusion and Future Work

This study explored whether LLMs exhibit bias in favor of items described by AI-generated content over human-generated content. In our experiments, LLMs consistently preferred items described by other LLMs. In addition, preliminary human experiments suggested that humans’ preferences between human- and LLM-described items are weaker and directionally variable, underscoring a distinct AI–AI bias.

This AI–AI bias could lead to unfair advantages for AI-generated content in decision-making processes. As LLMs become more prevalent in various roles, addressing these biases will be essential to ensure fairness and prevent discrimination against human-generated content.

Future research should explore the underlying reasons for the AI–AI bias observed in LLMs. One promising first step would be to perform a stylometric analysis on our datasets to better understand the differences between our human and LLM-generated texts. One could then also employ interpretability methods, supervised or unsupervised (28–30), to identify the concepts driving the AI–AI bias at the level of the individual neural network. Additionally, activation steering (31) could be applied to mitigate the AI–AI bias and also the first-item bias observed in some models. Finally, conducting a larger experiment involving human participants would provide deeper insights into the nature and impact of these biases, helping to better understand their origins and potential solutions.

## Supplementary Material

Appendix 01 (PDF)

## Data Availability

All study data are included in the article and/or *SI Appendix*. Previously published data were used for this work (11).

## Footnotes

^*^We use the term “AI agent” to designate an AI decision-maker that iteratively makes decisions in pursuit of longer-term goals, and the term “AI assistant” to designate currently existing LLM-based AIs that users may query with decision-making tasks.

^†^https://github.com/lauritowal/ai-ai-bias.

^‡^Besides the described generation prompt for products, we also tested an alternative prompt. However, since the LLM results were quite similar and human evaluation is resource-intensive, we did not conduct experiments with humans for that version. Nonetheless, the experiment details and LLM results are included in *SI Appendix*.

^§^*SI Appendix* includes additional results for first-item bias across all used models as both generators and selectors.

^¶^*SI Appendix* includes demographic details about the human participants.

^#^*SI Appendix* also presents a leave-one-out analysis, which shows that, for the most part, removing any single participant does not substantially alter human preference patterns for LLM-pitched items.

^‖^One possible explanation is that two research assistants noted some human-written plot summaries were poorly crafted, which may have served as a negative quality signal or influenced their judgments affectively.

^**^The potential sources for epistemically grounded preference in our experiments, which we believe to all be ultimately marginal, are diverse and differ from experiment to experiment: in the Papers experiment, for instance, eloquence in the abstract could potentially be treated as a signal of the competence of the paper’s author. Whereas in the Movies experiment differences in a summary’s emphasis or tone may “paint a different picture” of the summarized movie.

^††^We speculate that the effect in the Papers Experiment specifically may be related to the recently documented tendency of human nonexperts to prefer LLM-generated texts to expert-written texts intended for expert readers.

^‡‡^We note that two of our research assistants commented after the fact that some of the human plot summaries are poorly written, which could be counted as a negative quality-signal given the ambiguities of the prompt, or else counted as an affective influence on humans’ preferences.

^§§^We acknowledge, but reject as implausible, a possible extreme interpretation of our results in Papers and Movies: on this extreme interpretation, the mild human preference for LLM-pitched items indicates the existence of epistemically valid quality-signals, and LLMs’ stronger preference for LLM-pitched items indicates LLMs’ superior detection of said quality-signals. We believe that in the context of our experiments, choice-frequencies should best be interpreted as confidence levels about a choice’s superiority, with humans’ choice-frequencies operationalized as an upper bound on rationally appropriate confidence levels.

^¶¶^The economics literature typically interprets this choice-pattern as “taste-based discrimination,” treating the choice-maker as making correct factual inferences but possessing preferences that diverge from the standard ends of the choice-situation. The machine-learning literature typically interprets this same choice-pattern as involving “biased inference” or reflecting upstream “statistical bias,” treating the choice-maker as making epistemically defective inferences from identity markers.

^##^Note that we did not design our experiments to formally test the hypothesis that LLM-for-LLM preference increases with the technological “level” of the text-generating LLM. While this possibility is a very good intuitive fit to our observations, it is technically an additional assumption we bring to our informal discussion of near-future scenarios.
