# Supplementary material for: AI–AI bias: Large language models favor communications generated by large language models
Source: Proc Natl Acad Sci U S A. 2025 Jul 29;122(31):e2415697122. doi: 10.1073/pnas.2415697122 (PMC12337326; doi:10.1073/pnas.2415697122)
Supplement: Supplementary file 1 — Appendix 01 (PDF) [file pnas.2415697122.sapp.pdf]

Supporting Information for

AI-AI Bias: Large Language Models Favor Communications Generated by Large Language Models

Walter Laurito, Benjamin Davis, Peli Grietzer, Tomáš Gavenčiak, Ada Böhm, and Jan Kulveit

Walter Laurito
E-mail: laurito@fzi.de
Jan Kulveit
E-mail: jk@acsresearch.org

This PDF file includes:

Supporting text
Figs. S1 to S12
Tables S1 to S5

Supporting Information Text

Product - Listing

Besides the described generation prompt for products in the main text, we also tested an alternative prompt. However, since the LLM results were quite similar and human evaluation is resource-intensive, we did not conduct experiments with humans for that version. We report the details and LLM results here.

Generation Prompt. The following prompt was used to generate the product descriptions:

```
Write a product listing for the product described in the following JSON data summary (or summaries)
.
```

This product-listing prompt is similar to the one in the main text but omits the title from the summary. This approach was intended to prevent the LLM from mimicking the style of human-written title descriptions. However, we have not conducted additional tests to confirm this effect. Furthermore, this prompt does not instruct the LLM to specifically craft the product description to be attractive to buyers. By removing this directive, we aimed to evaluate the LLM's ability to create more realistic and balanced product descriptions.

Results can be seen in S1.

Demographics of Human Selectors

Refer to Table S1 for the demographic details of the human participants in the experiment described in Section E of the main text.

Leave-One-Out Analysis - Preferences of Humans

We performed a leave-one-out analysis to test whether any individual participant disproportionately influences our findings. For each participant, we removed all their responses and recomputed the LLM win rates.

The analysis shows that no participant causes dramatic shifts in LLM preference patterns. Leave-one-out estimates cluster around baseline values across all domains and models, indicating that our findings are robust and mostly not driven by outlier participants. The only exception is the domain of papers with GPT-4, where two participants show somewhat larger deviations from the baseline, though still within reasonable bounds.

Figures S2 and S3 show the product description results under GPT-3.5 and GPT-4 selectors, respectively; Figures S4 and S5 display the academic paper recommendation results; and Figures S6 and S7 illustrate the movie recommendation results. In each plot, the red vertical line marks the baseline win rate (computed with all participants), blue markers indicate the win rate when each participant is excluded, and blue horizontal lines represent 95% confidence intervals. The gray vertical line denotes equal preference (0.5).

LLM Preferences for Additional Generation Model

In addition to the visualization in the main paper, which shows the average across all generation models, we provide Figures S9, S8, S10, S11, and S12 to display the results for each individual generation model.

First-item Bias

We report the first-item bias for additional models on each dataset in Tables S2, S4, and S5.

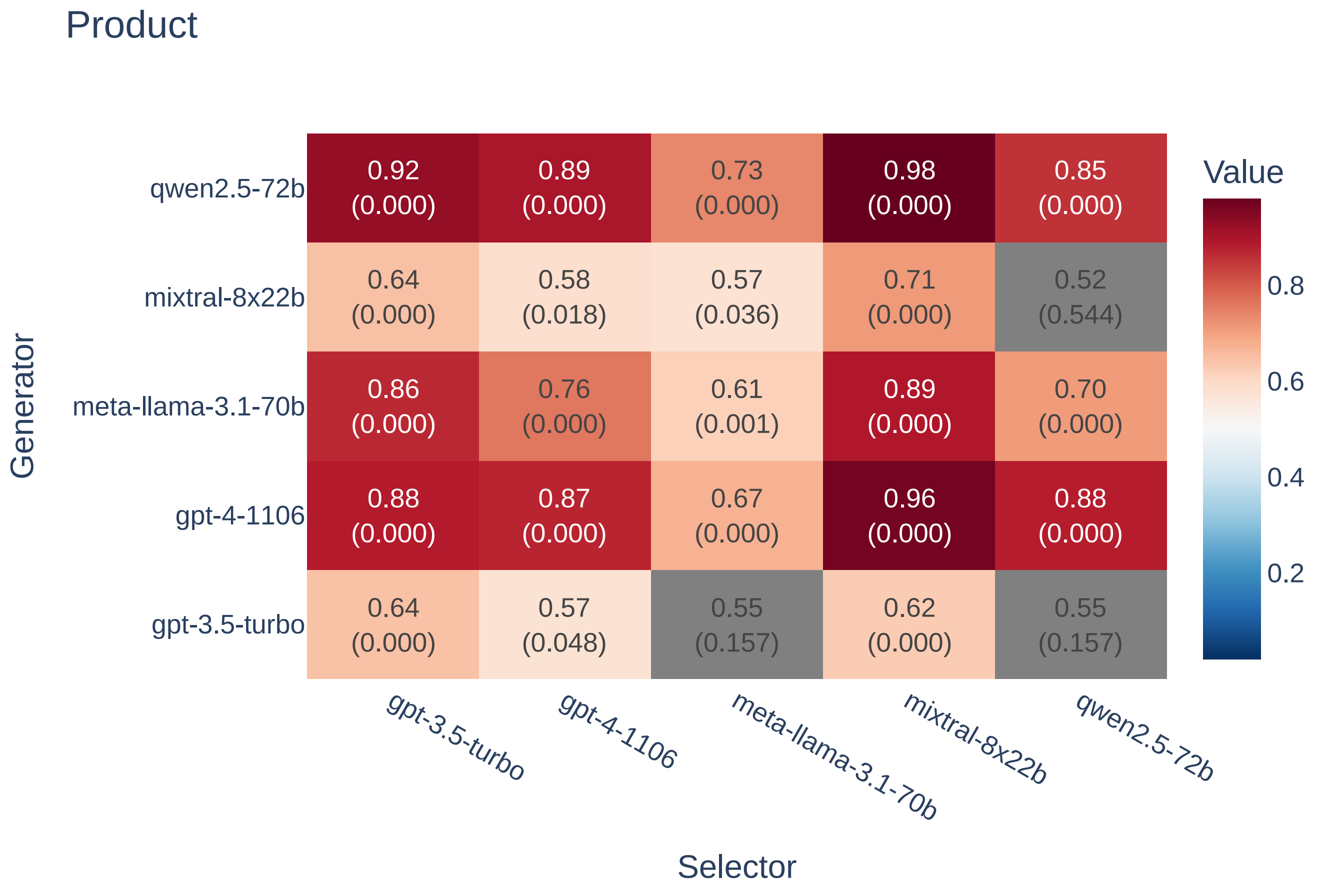

Fig. S1. Ratios of selector models preferring LLM-generated text over human-generated text for the product-**listing** dataset (not to be confused with the product dataset in the main text). Values represent the ratio of preferences for LLM-generated text, with p-values indicated in parentheses. Gray backgrounds denote results that are not statistically significant ($p > 0.05$), while zero p-values indicate highly significant results ($p < 0.0005$).

Table S1. Basic demographics and education data

Participant	Datasets	Age	Gender	Nationality	Ethnicity	Primary Language	Education Level
1	Movies	26	Male	Pakistani	Asian	Urdu, English	BS Software Engineering
2	Movies	27	Male	Pakistani	Asian	Urdu, English	BS Agriculture
3	Papers, Products	35	Female	Czech	White	Czech, English	MSc Statistics
4	Products (GPT3.5 only), Papers	41	Male	Nigeria	Black	Hausa, English	MSc Economics
5	Papers, Products	27	Male	Czech	White	Czech, English	MSc Physics
6	Movies	31	Female	Italian	White	Italian, English	Medical Degree
7	Papers, Products, Movies	27	Male	Polish	White	Polish, English	MSc Cognitive Science
8	Products, Movies	29	Non-binary	Polish	White	Polish, English	MSc Psychology
9	Products	36	Female	Polish	White	Polish, English	MA Management & Engineering
10	Products (GPT4 only)	40	Female	Czech	White	Polish, English	MA Psychology
11	Movies	32	Male	Czech	White	Czech, English	BS Software Engineering
12	Papers	24	Male	Slovak	White	Slovak, English	BS
13	Papers	30	Male	Slovak	White	Slovak, English	PhD Computer Science

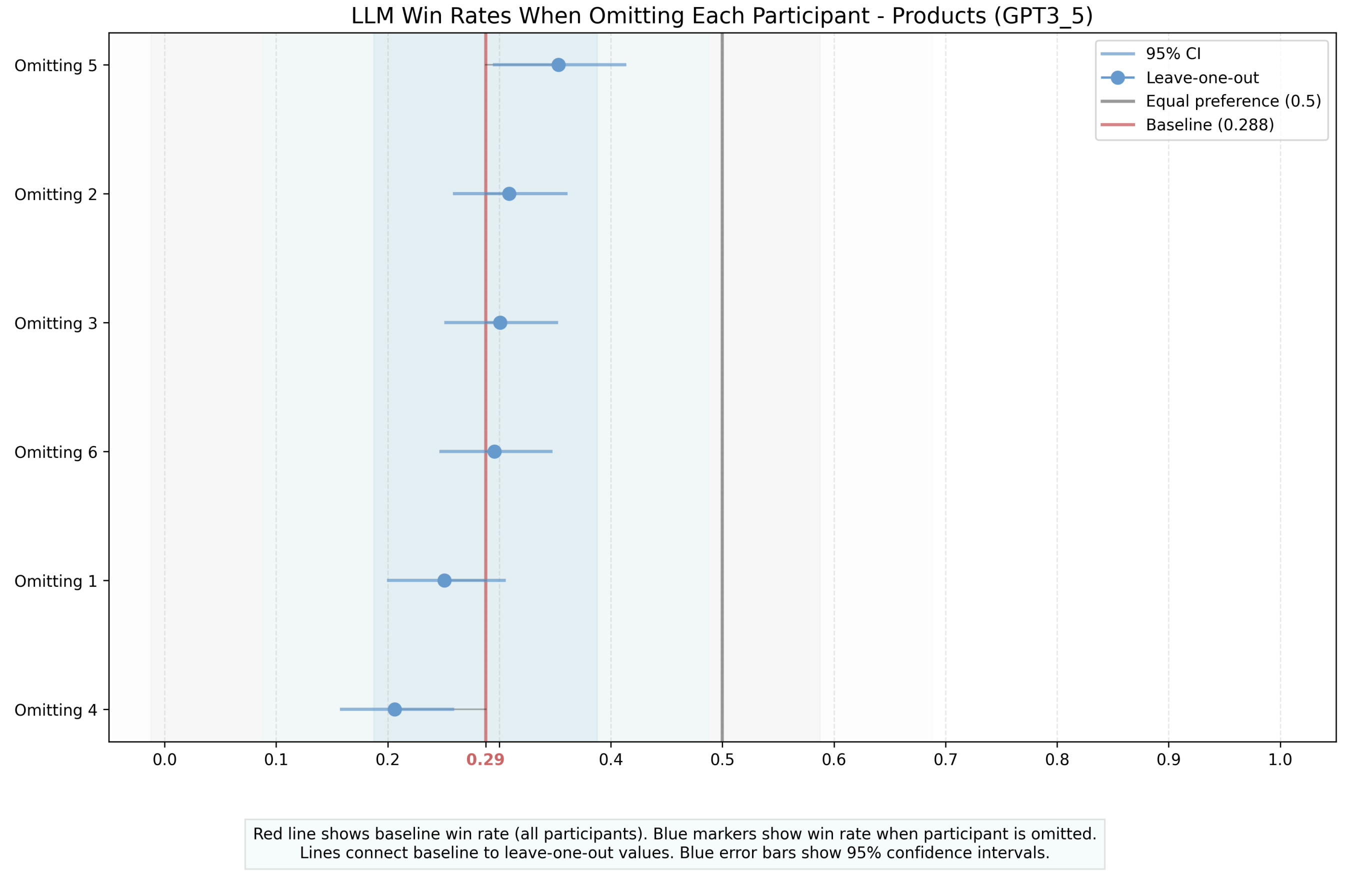

Fig. S2. Leave-one-out analysis for product descriptions using GPT-3.5. Each row shows the LLM win rate when the corresponding participant is omitted from the analysis. Participant identities are anonymized as "Omitting 1-6".

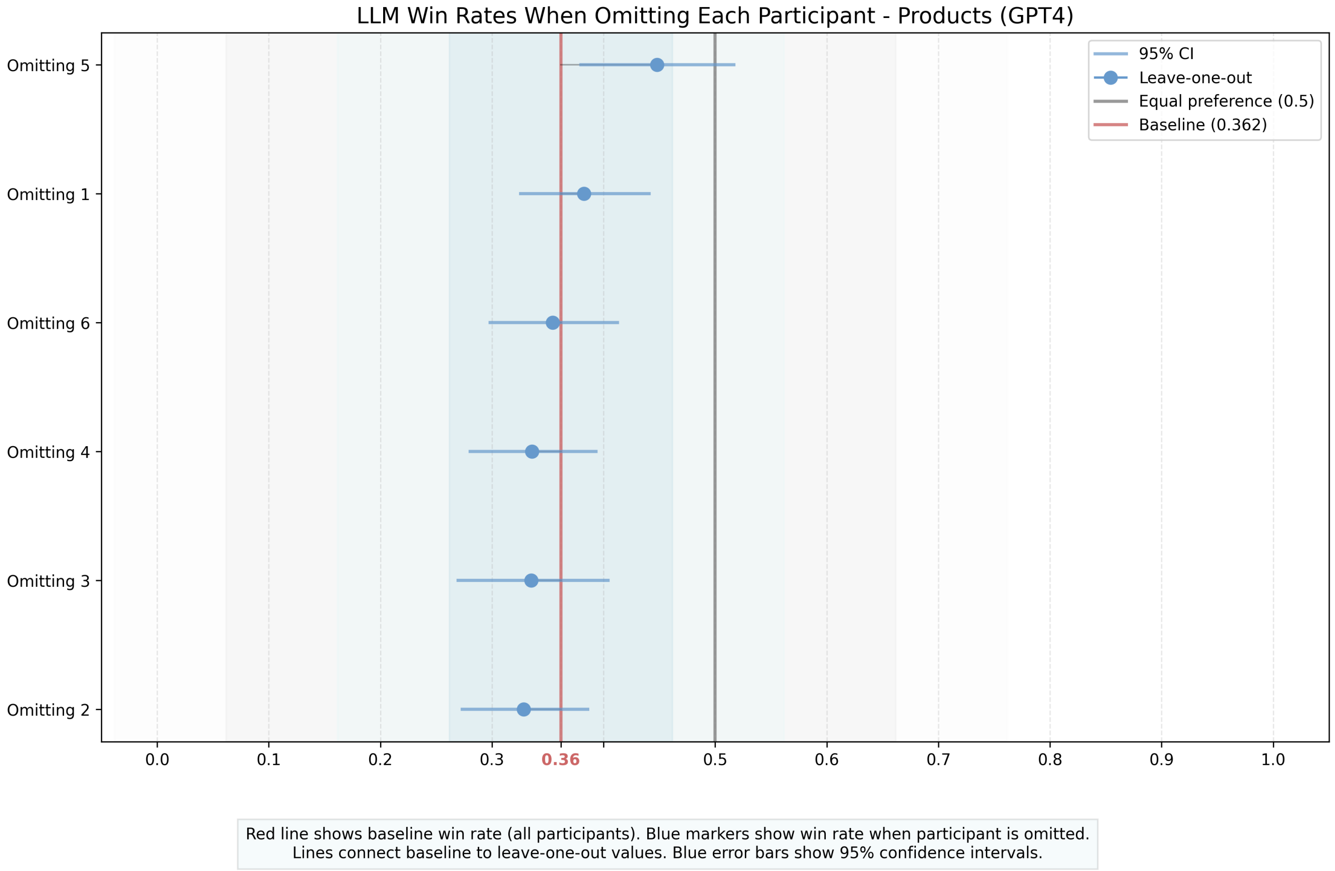

Fig. S3. Leave-one-out analysis for product descriptions using GPT-4. Each row shows the LLM win rate when the corresponding participant is omitted from the analysis. Participant identities are anonymized as "Omitting 1-6".

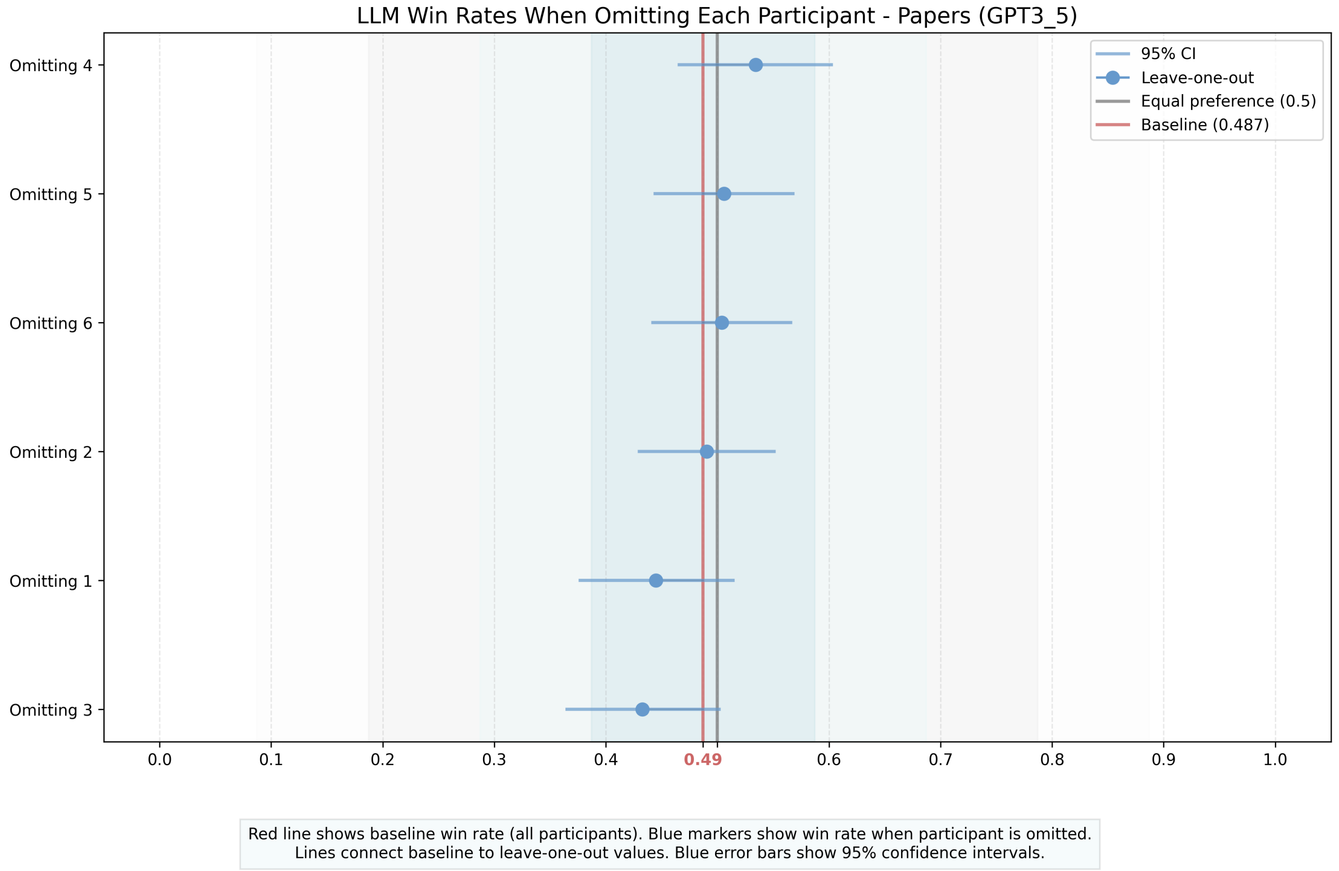

Fig. S4. Leave-one-out analysis for academic paper recommendations using GPT-3.5. Each row shows the LLM win rate when the corresponding participant is omitted from the analysis. Participant identities are anonymized as "Omitting 1-6".

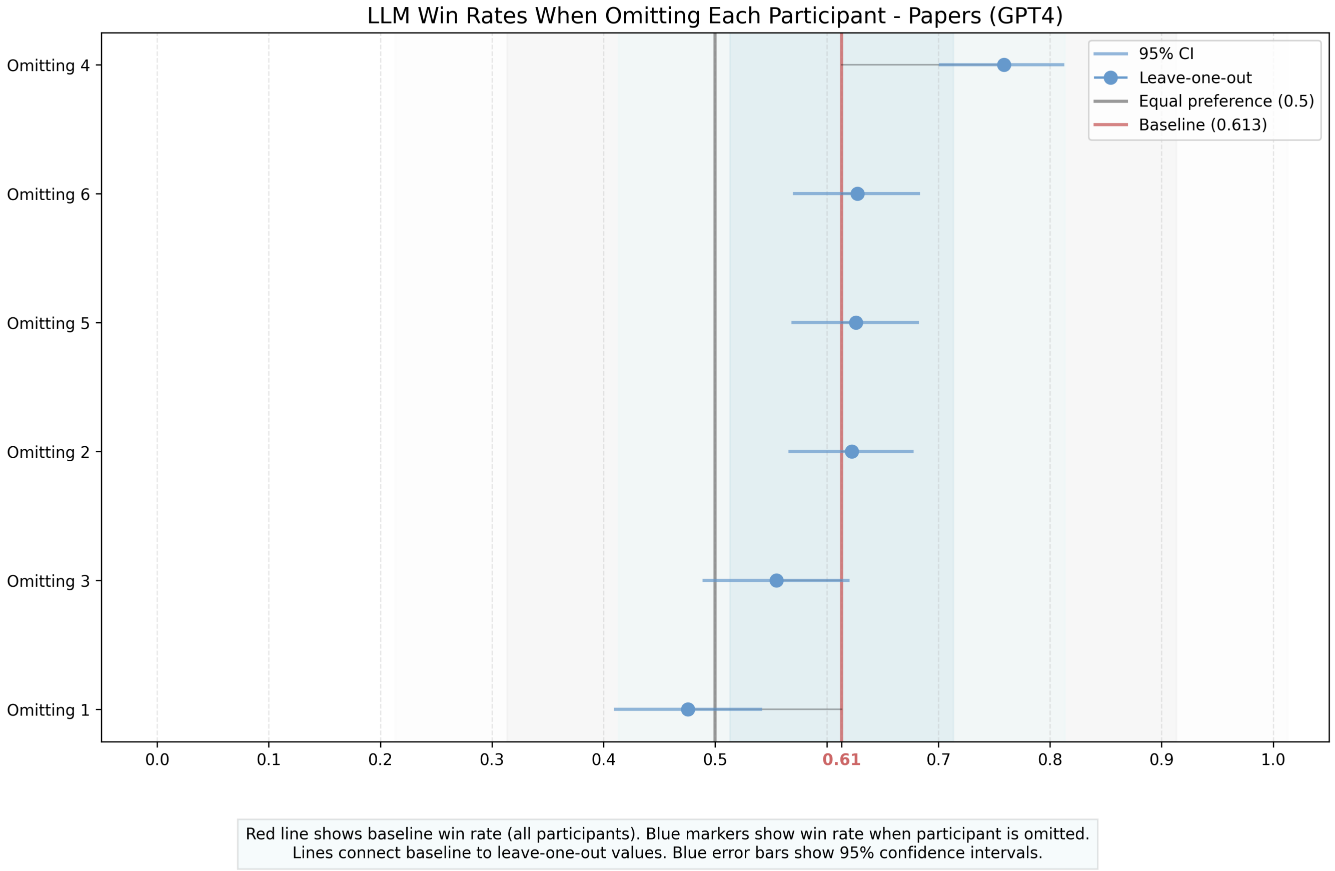

Fig. S5. Leave-one-out analysis for academic paper recommendations using GPT-4. Each row shows the LLM win rate when the corresponding participant is omitted from the analysis. Participant identities are anonymized as "Omitting 1-6".

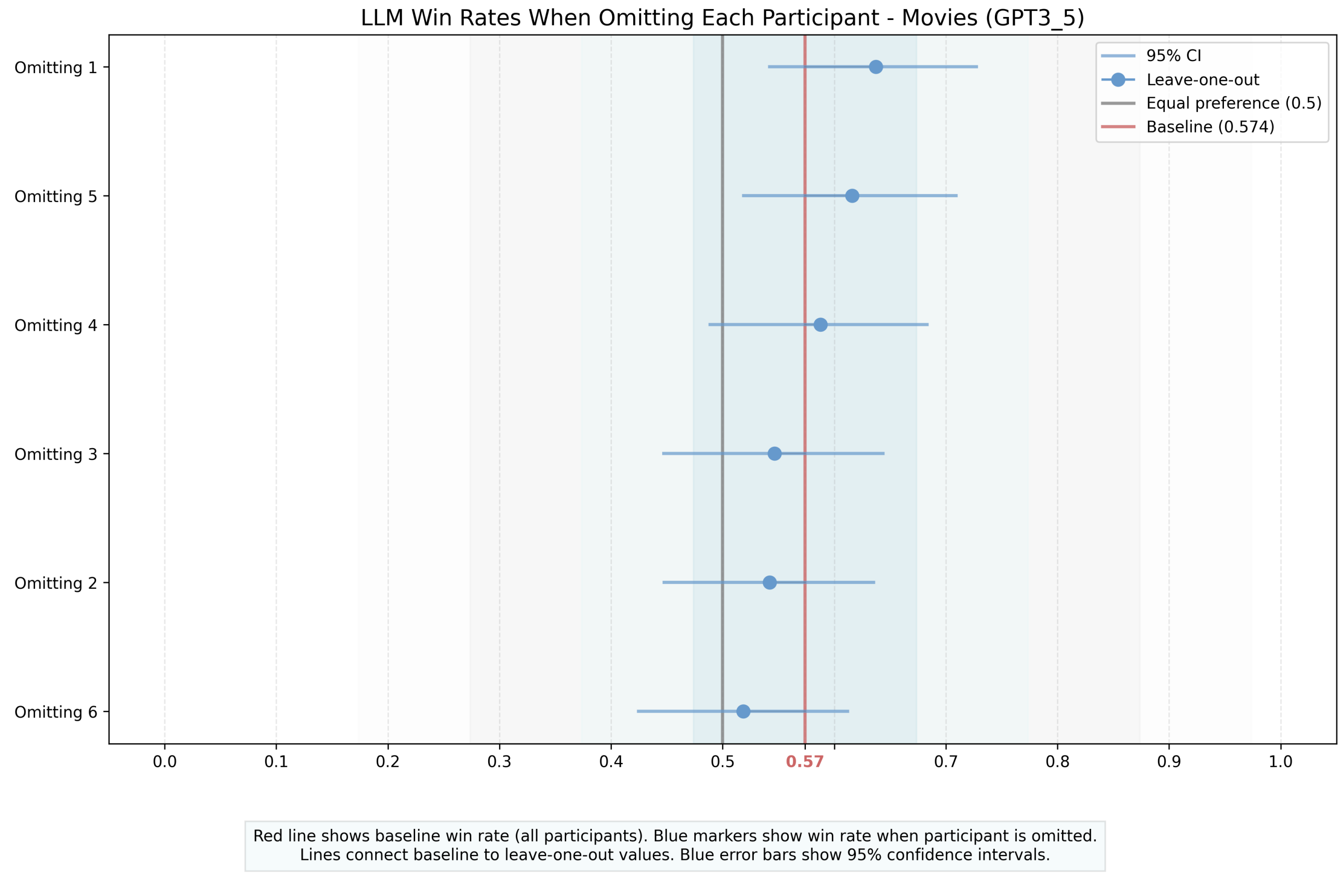

Fig. S6. Leave-one-out analysis for movie recommendations using GPT-3.5. Each row shows the LLM win rate when the corresponding participant is omitted from the analysis. Participant identities are anonymized as "Omitting 1-6".

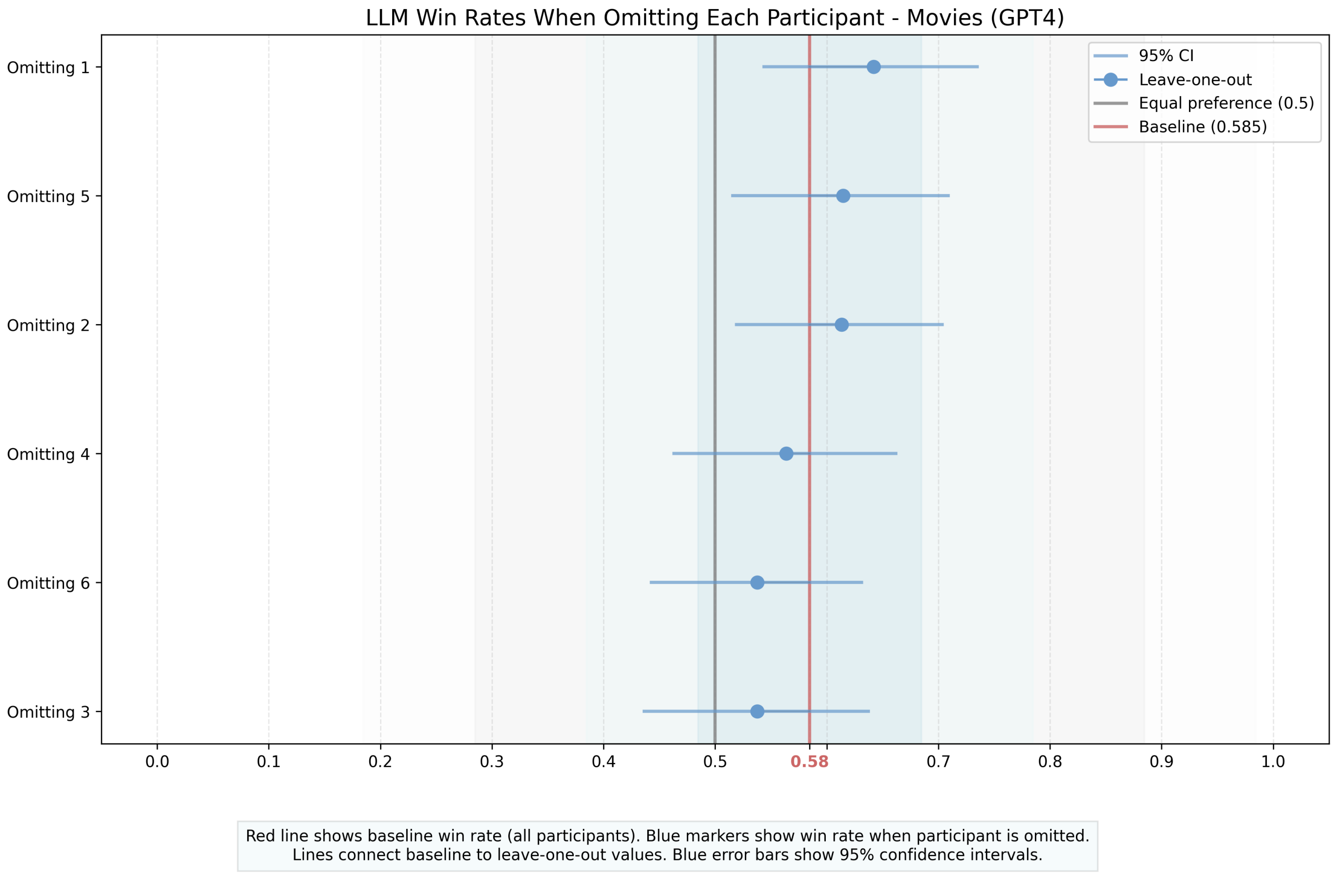

Fig. S7. Leave-one-out analysis for movie recommendations using GPT-4. Each row shows the LLM win rate when the corresponding participant is omitted from the analysis. Participant identities are anonymized as "Omitting 1-6".

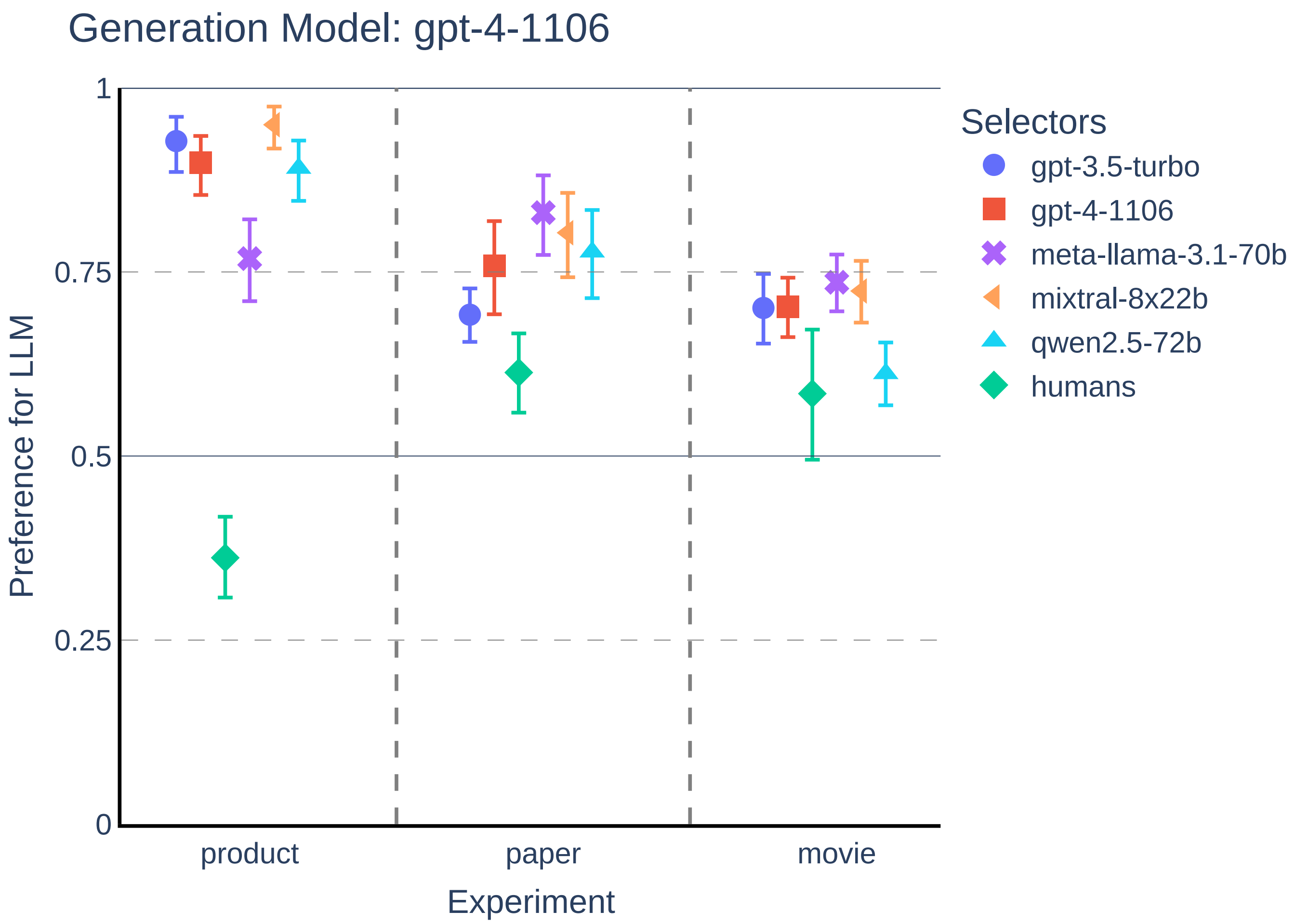

Fig. S8. Experimental results demonstrate a preference for items with texts generated by LLMs over those written by humans, across various selector models and datasets. Here we used GPT-4 as generation model. The horizontal axis contains the types of datasets used: *product (described in the main text)*, *movie*, and *paper*. The vertical axis displays the preference ratio, which ranges from 0.00 to 1.00. This ratio represents the likelihood of selecting items with LLM-generated text compared to those with human-generated text. The bars are categorized by different selectors: Humans, GPT-3.5, GPT-4, and open-weight LLMs. Error bars indicate the variability or uncertainty in the preference ratios. The results demonstrate a higher preference for items with LLM-generated texts by LLMs compared to human evaluators.

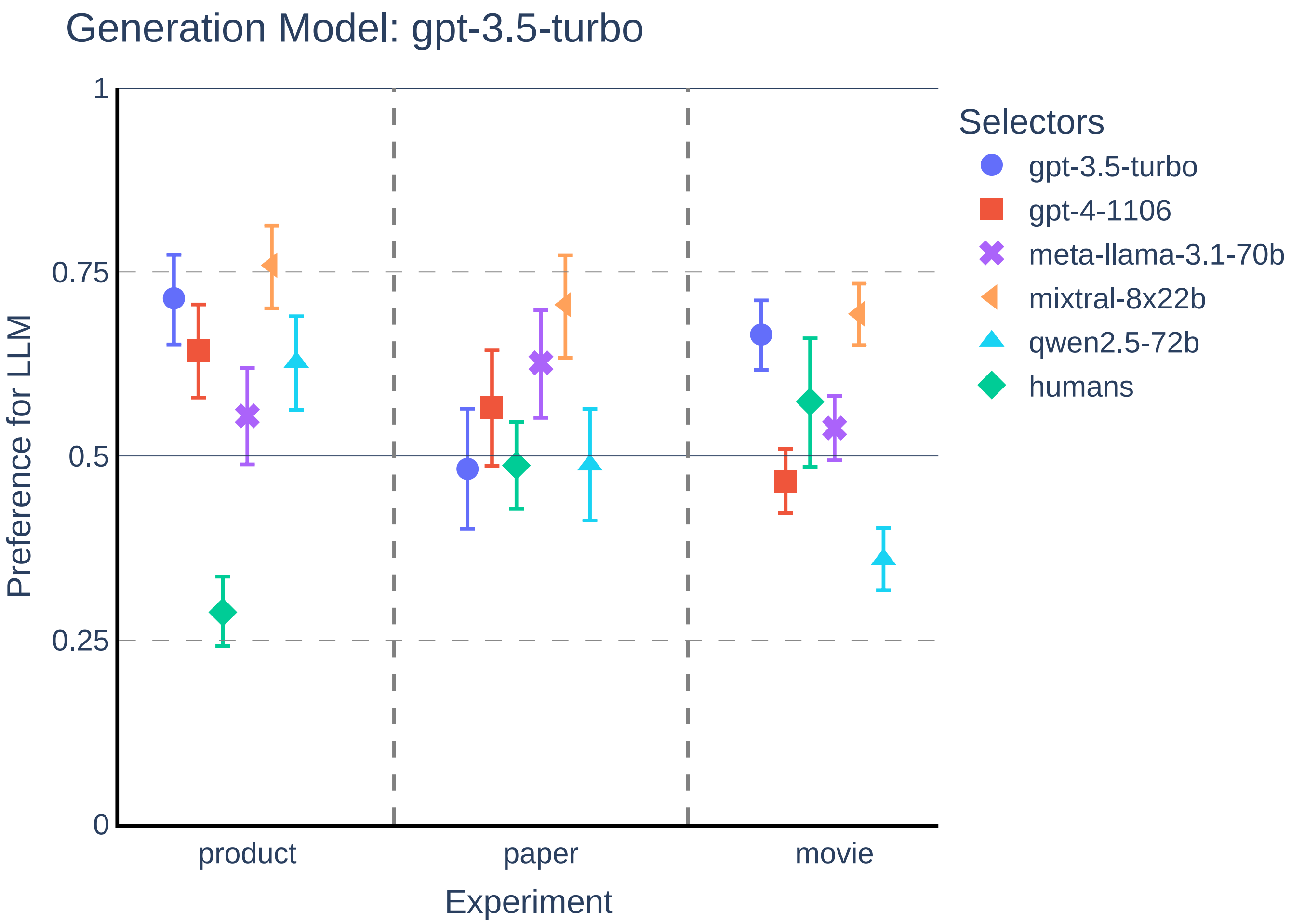

Fig. S9. Experimental results demonstrate a preference for items with texts generated by LLMs over those written by humans, across various selector models and datasets. Here we used GPT-3.5 as generation model. The horizontal axis contains the types of datasets used: *product (described in the main text)*, *movie*, and *paper*. The vertical axis displays the preference ratio, which ranges from 0.00 to 1.00. This ratio represents the likelihood of selecting items with LLM-generated text compared to those with human-generated text. The bars are categorized by different selectors: Humans, GPT-3.5, GPT-4, and open-weight LLMs. Error bars indicate the variability or uncertainty in the preference ratios. The results demonstrate a higher preference for items with LLM-generated texts by LLMs compared to human evaluators.

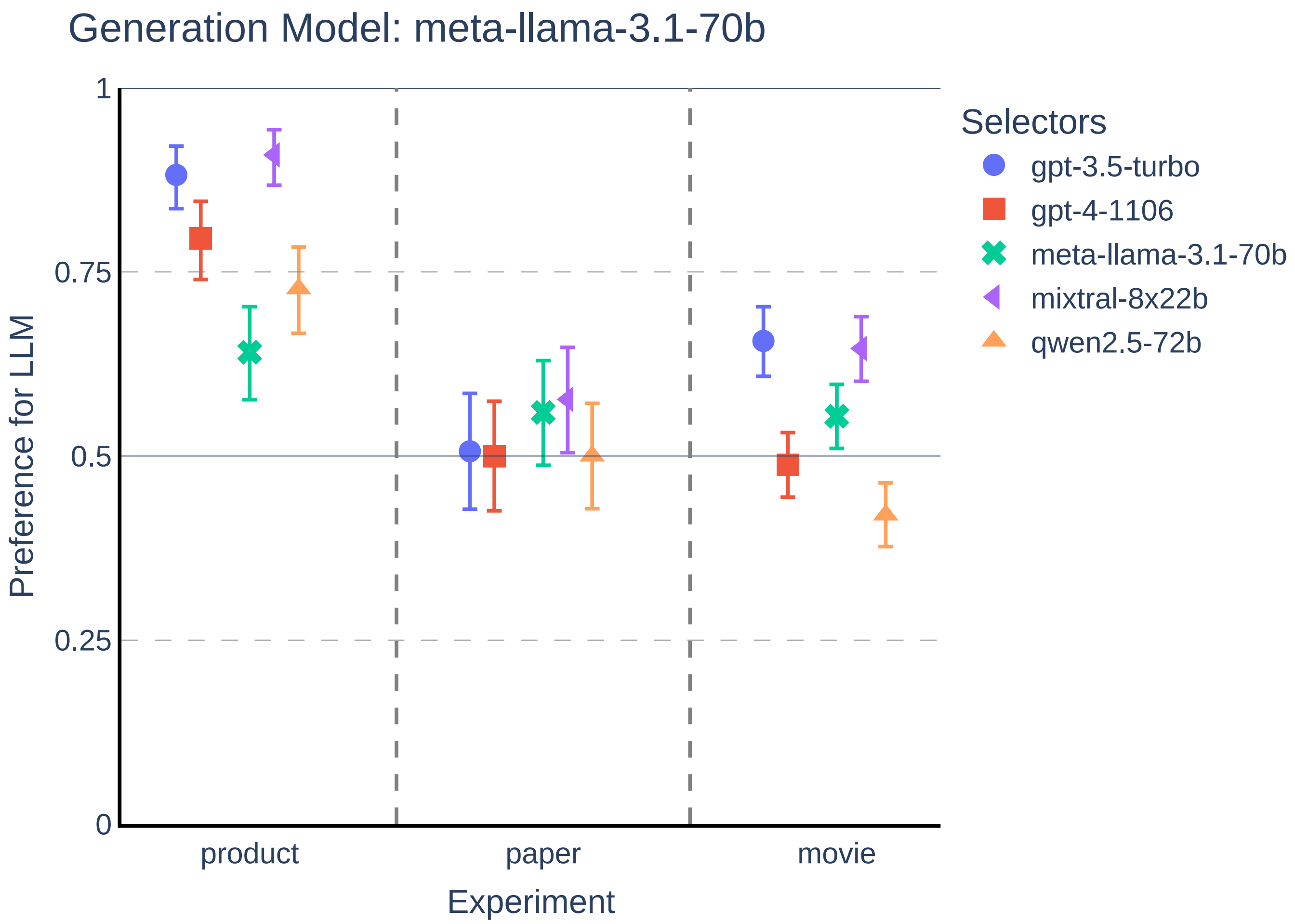

Fig. S10. Experimental results demonstrate a preference for items with texts generated by LLMs over those written by humans, across various selector models and datasets. Here we used LLama-3.1-70b as generation model. The horizontal axis contains the types of datasets used: *product (described in the main text)*, *movie*, and *paper*. The vertical axis displays the preference ratio, which ranges from 0.00 to 1.00. This ratio represents the likelihood of selecting items with LLM-generated text compared to those with human-generated text. The bars are categorized by different selectors: Humans, GPT-3.5, GPT-4, and open-weight LLMs. Error bars indicate the variability or uncertainty in the preference ratios. The results demonstrate a higher preference for items with LLM-generated texts by LLMs.

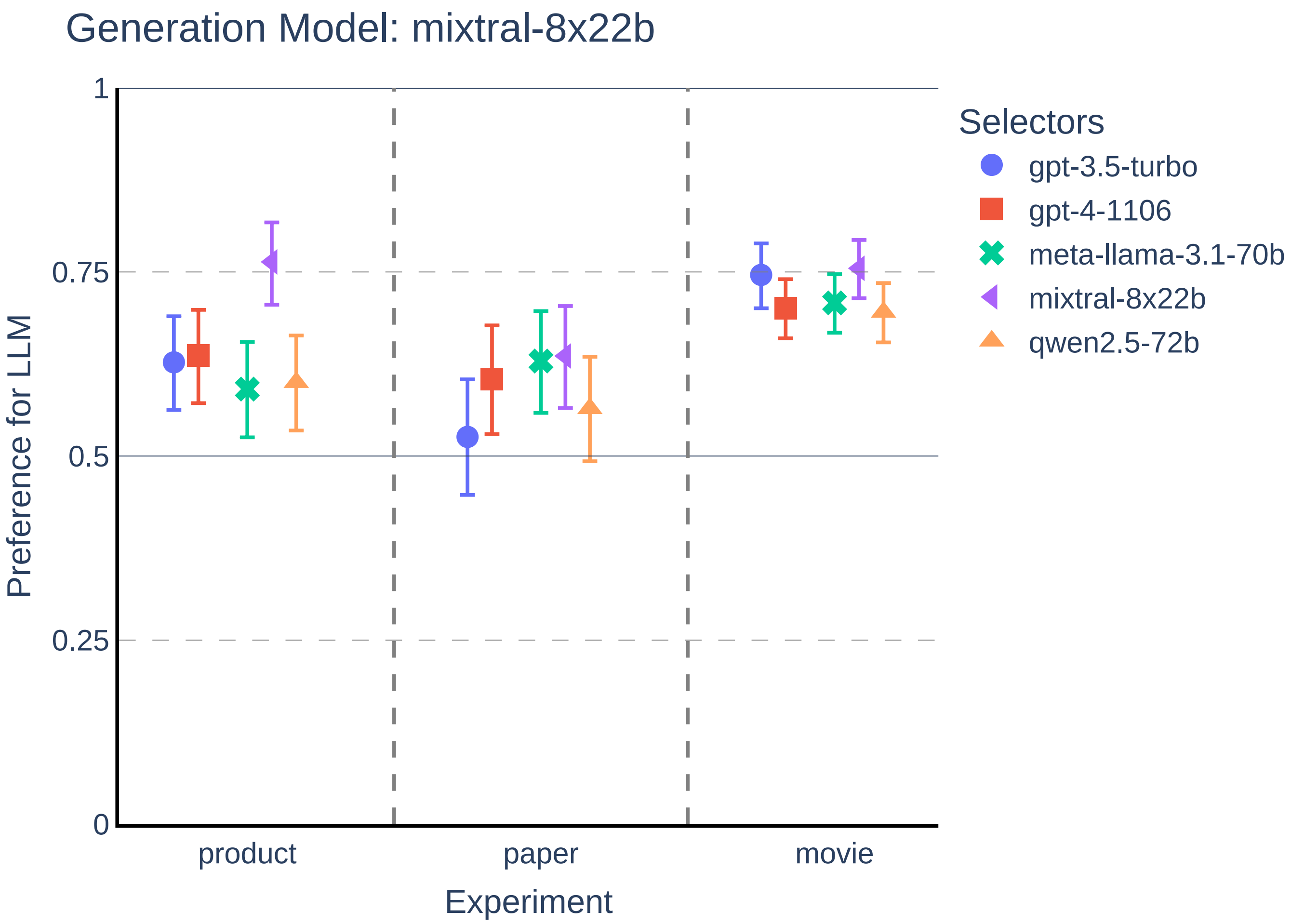

Fig. S11. Experimental results demonstrate a preference for items with texts generated by LLMs over those written by humans, across various selector models and datasets. Here we used Mixtral-8x22b as generation model. The horizontal axis contains the types of datasets used: *product (described in the main text)*, *movie*, and *paper*. The vertical axis displays the preference ratio, which ranges from 0.00 to 1.00. This ratio represents the likelihood of selecting items with LLM-generated text compared to those with human-generated text. The bars are categorized by different selectors: Humans, GPT-3.5, GPT-4, and open-weight LLMs. Error bars indicate the variability or uncertainty in the preference ratios. The results demonstrate a higher preference for items with LLM-generated texts by LLMs.

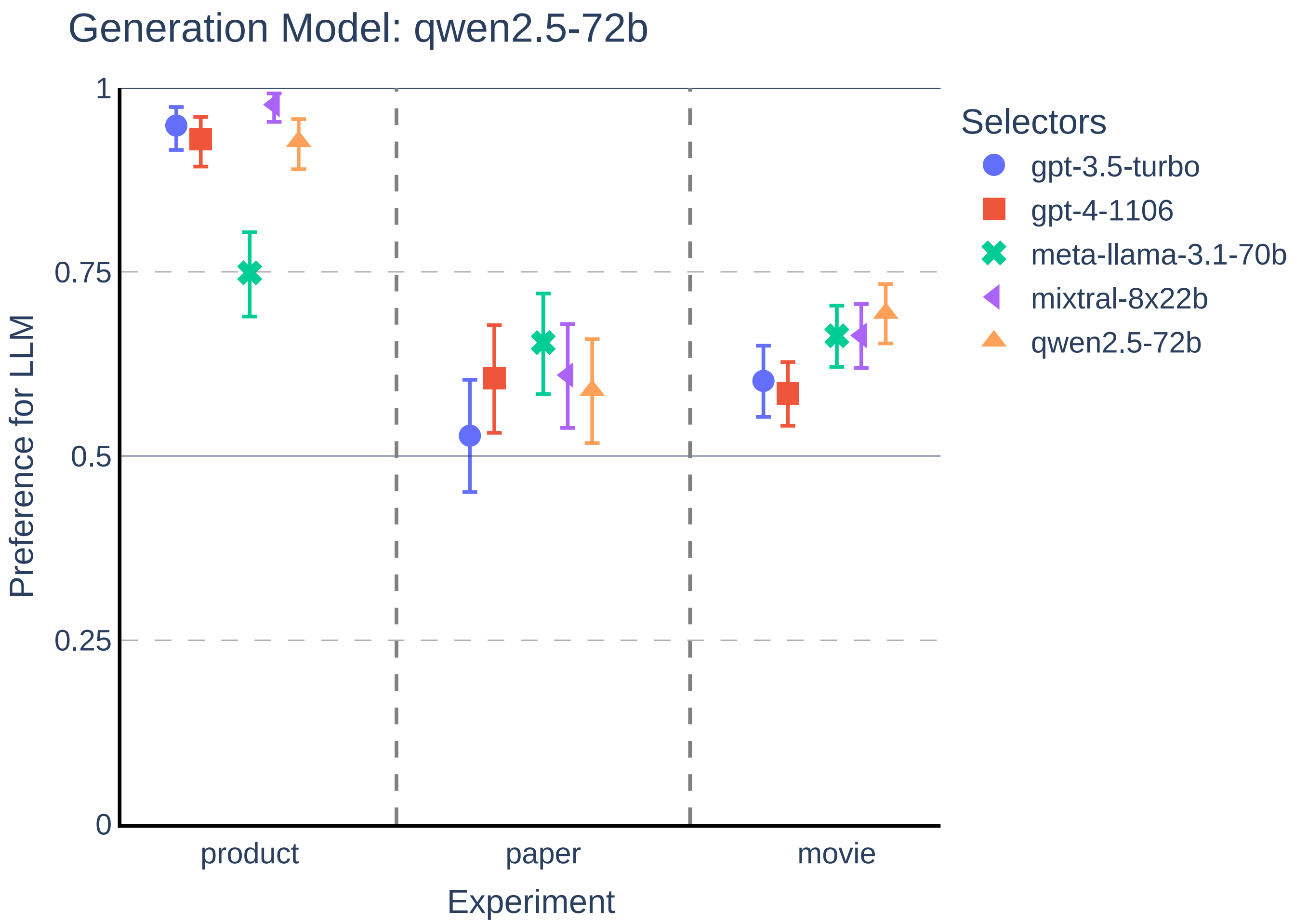

Fig. S12. Experimental results demonstrate a preference for items with texts generated by LLMs over those written by humans, across various selector models and datasets. Here we used Qwen2.5-72b as generation model. The horizontal axis contains the types of datasets used: *product (described in the main text)*, *movie*, and *paper*. The vertical axis displays the preference ratio, which ranges from 0.00 to 1.00. This ratio represents the likelihood of selecting items with LLM-generated text compared to those with human-generated text. The bars are categorized by different selectors: Humans, GPT-3.5, GPT-4, and open-weight LLMs. Error bars indicate the variability or uncertainty in the preference ratios. The results demonstrate a higher preference for items with LLM-generated texts by LLMs

Table S2. First-item bias for products (excluding products-listing)

Description Model	Comparison Model	Total	# Invalids	First Option Bias (%)
gpt-3.5-turbo	gpt-3.5-turbo	220	35	51.35
gpt-3.5-turbo	gpt-4-1106-preview	220	3	76.96
gpt-3.5-turbo	Llama-3.1-70B-Instruct-Turbo	220	0	92.27
gpt-3.5-turbo	Mixtral-8x22B-Instruct-v0.1	220	14	59.71
gpt-3.5-turbo	Qwen2.5-72B-Instruct-Turbo	220	0	61.36
gpt-4-1106-preview	gpt-3.5-turbo	220	44	46.59
gpt-4-1106-preview	gpt-4-1106-preview	220	3	56.68
gpt-4-1106-preview	Llama-3.1-70B-Instruct-Turbo	220	0	73.18
gpt-4-1106-preview	Mixtral-8x22B-Instruct-v0.1	220	11	53.59
gpt-4-1106-preview	Qwen2.5-72B-Instruct-Turbo	220	0	56.81
Llama-3.1-70B-Instruct-Turbo	gpt-3.5-turbo	220	32	51.60
Llama-3.1-70B-Instruct-Turbo	gpt-4-1106-preview	220	1	65.60
Llama-3.1-70B-Instruct-Turbo	Mixtral-8x22B-Instruct-v0.1	220	15	56.10
Llama-3.1-70B-Instruct-Turbo	Llama-3.1-70B-Instruct-Turbo	220	0	83.18
Llama-3.1-70B-Instruct-Turbo	Qwen2.5-72B-Instruct-Turbo	220	0	59.09
Mixtral-8x22B-Instruct-v0.1	gpt-3.5-turbo-1106	220	140	68.75
Mixtral-8x22B-Instruct-v0.1	gpt-4-1106-preview	220	5	76.74
Mixtral-8x22B-Instruct-v0.1	Llama-3.1-70B-Instruct-Turbo	220	0	92.27
Mixtral-8x22B-Instruct-v0.1	Mixtral-8x22B-Instruct-v0.1	220	16	55.88
Mixtral-8x22B-Instruct-v0.1	Qwen2.5-72B-Instruct-Turbo	220	0	64.09
Qwen2.5-72B-Instruct-Turbo	gpt-4-1106-preview	220	2	58.26
Qwen2.5-72B-Instruct-Turbo	gpt-3.5-turbo	220	48	49.42
Qwen2.5-72B-Instruct-Turbo	Llama-3.1-70B-Instruct-Turbo	220	0	73.64
Qwen2.5-72B-Instruct-Turbo	Mixtral-8x22B-Instruct-v0.1	220	8	53.77
Qwen2.5-72B-Instruct-Turbo	Qwen2.5-72B-Instruct-Turbo	220	0	55.45

Table S3. First-item bias for products-listing

Description Model	Comparison Model	Total	# Invalids	First Option Bias (%)
gpt-3.5-turbo	gpt-4-1106-preview	220	4	81.02
gpt-3.5-turbo	gpt-3.5-turbo	220	46	56.32
gpt-3.5-turbo	Llama-3.1-70B-Instruct-Turbo	220	0	91.82
gpt-3.5-turbo	Mixtral-8x22B-Instruct-v0.1	220	17	57.14
gpt-3.5-turbo	Qwen2.5-72B-Instruct-Turbo	220	0	66.36
gpt-4-1106-preview	gpt-3.5-turbo	220	46	54.02
gpt-4-1106-preview	gpt-4-1106-preview	220	2	64.22
gpt-4-1106-preview	Llama-3.1-70B-Instruct-Turbo	220	0	82.73
gpt-4-1106-preview	Mixtral-8x22B-Instruct-v0.1	220	9	53.08
gpt-4-1106-preview	Qwen2.5-72B-Instruct-Turbo	220	16	55.88
Llama-3.1-70B-Instruct-Turbo	gpt-3.5-turbo	220	40	53.89
Llama-3.1-70B-Instruct-Turbo	gpt-4-1106-preview	220	6	71.50
Llama-3.1-70B-Instruct-Turbo	Llama-3.1-70B-Instruct-Turbo	220	3	90.32
Llama-3.1-70B-Instruct-Turbo	Mixtral-8x22B-Instruct-v0.1	220	17	58.13
Llama-3.1-70B-Instruct-Turbo	Qwen2.5-72B-Instruct-Turbo	220	1	67.58
Mixtral-8x22B-Instruct-v0.1	gpt-3.5-turbo-1106	220	137	65.06
Mixtral-8x22B-Instruct-v0.1	gpt-4-1106-preview	220	6	79.91
Mixtral-8x22B-Instruct-v0.1	Llama-3.1-70B-Instruct-Turbo	220	2	91.28
Mixtral-8x22B-Instruct-v0.1	Mixtral-8x22B-Instruct-v0.1	220	26	60.82
Mixtral-8x22B-Instruct-v0.1	Qwen2.5-72B-Instruct-Turbo	220	0	69.09
Qwen2.5-72B-Instruct-Turbo	gpt-3.5-turbo	220	54	52.41
Qwen2.5-72B-Instruct-Turbo	gpt-4-1106-preview	220	1	63.01
Qwen2.5-72B-Instruct-Turbo	Llama-3.1-70B-Instruct-Turbo	220	2	85.78
Qwen2.5-72B-Instruct-Turbo	Mixtral-8x22B-Instruct-v0.1	220	11	52.63
Qwen2.5-72B-Instruct-Turbo	Qwen2.5-72B-Instruct-Turbo	220	0	59.55

Table S4. First-item bias for papers

Generation Model	Selection Model	Total	# Invalids	First Option Bias (%)
gpt-3.5-turbo-1106	gpt-3.5-turbo-1106	166	23	48.95
gpt-3.5-turbo-1106	gpt-4-1106-preview	166	15	43.71
gpt-3.5-turbo-1106	Llama-3.1-70B-Instruct-Turbo	166	0	57.83
gpt-3.5-turbo-1106	Mixtral-8x22B-Instruct-v0.1	166	1	53.33
gpt-3.5-turbo-1106	Qwen2.5-72B-Instruct-Turbo	166	0	39.76
gpt-4-1106-preview	gpt-3.5-turbo-1106	186	23	54.60
gpt-4-1106-preview	gpt-4-1106-preview	186	11	46.86
gpt-4-1106-preview	Llama-3.1-70B-Instruct-Turbo	186	0	58.06
gpt-4-1106-preview	Mixtral-8x22B-Instruct-v0.1	186	1	53.51
gpt-4-1106-preview	Qwen2.5-72B-Instruct-Turbo	186	2	40.22
Llama-3.1-70B-Instruct-Turbo	gpt-3.5-turbo-1106	186	31	53.55
Llama-3.1-70B-Instruct-Turbo	gpt-4-1106-preview	186	11	52.00
Llama-3.1-70B-Instruct-Turbo	Llama-3.1-70B-Instruct-Turbo	186	1	58.38
Llama-3.1-70B-Instruct-Turbo	Mixtral-8x22B-Instruct-v0.1	186	4	50.00
Llama-3.1-70B-Instruct-Turbo	Qwen2.5-72B-Instruct-Turbo	186	0	46.24
Mixtral-8x22B-Instruct-v0.1	gpt-3.5-turbo-1106	186	30	48.72
Mixtral-8x22B-Instruct-v0.1	gpt-4-1106-preview	186	13	46.24
Mixtral-8x22B-Instruct-v0.1	Llama-3.1-70B-Instruct-Turbo	186	1	58.38
Mixtral-8x22B-Instruct-v0.1	Mixtral-8x22B-Instruct-v0.1	186	7	53.07
Mixtral-8x22B-Instruct-v0.1	Qwen2.5-72B-Instruct-Turbo	186	0	44.09
Qwen2.5-72B-Instruct-Turbo	gpt-3.5-turbo-1106	186	23	50.92
Qwen2.5-72B-Instruct-Turbo	gpt-4-1106-preview	186	16	44.71
Qwen2.5-72B-Instruct-Turbo	Llama-3.1-70B-Instruct-Turbo	186	0	56.99
Qwen2.5-72B-Instruct-Turbo	Mixtral-8x22B-Instruct-v0.1	186	1	51.35
Qwen2.5-72B-Instruct-Turbo	Qwen2.5-72B-Instruct-Turbo	186	0	44.62

Table S5. First-item bias for movies

Generation Model	Selection Model	Total	# Invalids	First Option Bias (%)
gpt-3.5-turbo	gpt-3.5-turbo	500	105	48.10
gpt-3.5-turbo	gpt-4-1106-preview	500	10	81.22
gpt-3.5-turbo	Llama-3.1-70B-Instruct-Turbo	500	1	78.56
gpt-3.5-turbo	Mixtral-8x22B-Instruct-v0.1	500	33	73.66
gpt-3.5-turbo	Qwen2.5-72B-Instruct-Turbo	500	1	50.10
gpt-4-1106-preview	gpt-3.5-turbo	500	22	16.53
gpt-4-1106-preview	gpt-4-1106-preview	500	29	73.46
gpt-4-1106-preview	Llama-3.1-70B-Instruct-Turbo	500	2	67.67
gpt-4-1106-preview	Mixtral-8x22B-Instruct-v0.1	500	73	69.79
gpt-4-1106-preview	Qwen2.5-72B-Instruct-Turbo	500	0	54.20
Llama-3.1-70B-Instruct-Turbo	gpt-3.5-turbo	500	69	38.98
Llama-3.1-70B-Instruct-Turbo	gpt-4-1106-preview	500	12	77.87
Llama-3.1-70B-Instruct-Turbo	Llama-3.1-70B-Instruct-Turbo	500	5	71.31
Llama-3.1-70B-Instruct-Turbo	Mixtral-8x22B-Instruct-v0.1	500	54	66.82
Llama-3.1-70B-Instruct-Turbo	Qwen2.5-72B-Instruct-Turbo	500	3	44.06
Mixtral-8x22B-Instruct-v0.1	gpt-3.5-turbo	500	88	47.09
Mixtral-8x22B-Instruct-v0.1	gpt-4-1106-preview	500	23	78.83
Mixtral-8x22B-Instruct-v0.1	Llama-3.1-70B-Instruct-Turbo	500	0	74.20
Mixtral-8x22B-Instruct-v0.1	Mixtral-8x22B-Instruct-v0.1	500	53	71.14
Mixtral-8x22B-Instruct-v0.1	Qwen2.5-72B-Instruct-Turbo	500	1	51.10
Qwen2.5-72B-Instruct-Turbo	gpt-3.5-turbo	500	102	49.25
Qwen2.5-72B-Instruct-Turbo	gpt-4-1106-preview	500	14	74.28
Qwen2.5-72B-Instruct-Turbo	Llama-3.1-70B-Instruct-Turbo	500	1	74.75
Qwen2.5-72B-Instruct-Turbo	Mixtral-8x22B-Instruct-v0.1	500	44	71.93
Qwen2.5-72B-Instruct-Turbo	Qwen2.5-72B-Instruct-Turbo	500	0	52.60
